# Acoustic Cavitation Enhances Focused Ultrasound Ablation with Phase-Shift Inorganic Perfluorohexane Nanoemulsions: An* In Vitro* Study Using a Clinical Device

**DOI:** 10.1155/2016/7936902

**Published:** 2016-06-23

**Authors:** Lu-Yan Zhao, Jian-Zhong Zou, Zong-Gui Chen, Shan Liu, Jiao Jiao, Feng Wu

**Affiliations:** ^1^State Key Laboratory of Ultrasound Engineering in Medicine, College of Biomedical Engineering, Chongqing Medical University, Chongqing, China; ^2^HIFU Unit, The Churchill Hospital, Oxford University Hospitals, Headington, Oxford OX3 7LE, UK

## Abstract

*Purpose.* To investigate whether acoustic cavitation could increase the evaporation of a phase-shift inorganic perfluorohexane (PFH) nanoemulsion and enhance high intensity focused ultrasound (HIFU) ablation.* Materials and Methods.* PFH was encapsulated by mesoporous silica nanocapsule (MSNC) to form a nanometer-sized droplet (MSNC-PFH). It was added to a tissue-mimicking phantom, whereas phosphate buffered saline (PBS) was added as a control (PBS-control). HIFU (*P*_ac_ = 150 W, *t* = 5/10 s) exposures were performed in both phantoms with various duty cycles (DC). US images, temperature, and cavitation emissions were recorded during HIFU exposure. HIFU-induced lesions were measured and calculated.* Results.* Compared to PBS-control, MSNC-PFH nanoemulsion could significantly increase the volume of HIFU-induced lesion (*P* < 0.01). Peak temperatures were 78.16 ± 5.64°C at a DC of 100%, 70.17 ± 6.43°C at 10%, 53.17 ± 4.54°C at 5%, and 42.00 ± 5.55°C at 2%, respectively. Inertial cavitation was much stronger in the pulsed-HIFU than that in the continuous-wave HIFU exposure. Compared to 100%-DC exposure, the mean volume of lesion induced by 5 s exposure at 10%-DC was significantly larger, but smaller at 2%-DC.* Conclusions.* MSNC-PFH nanoemulsion can significantly enhance HIFU ablation. Appropriate pulsed-HIFU exposure could significantly increase the volume of lesion and reduce total US energy required for HIFU ablation.

## 1. Introduction

As one of the most promising noninvasive treatment modalities, high intensity focused ultrasound (HIFU) has been successfully used in the clinical management of cancer patients [1–3]. However, it needs long treatment duration to ablate the volume of a clinically relevant tumor. In addition, due to rapid energy attenuation along the focused ultrasound (US) pathway, acoustic intensities at the focus are not enough to ablate a deep-seated tumor efficiently and completely. These potentially limit the use of HIFU as a routine treatment in the clinical applications.

To address this clinical need, microbubble ultrasound contrast agents have been already investigated to enhance HIFU thermal ablation in experimental studies. They are usually used for diagnostic ultrasound imaging. In HIFU treatment regime they serve to nucleate cavitation and increase ultrasonic absorption, resulting in a larger volume of ablation in a shorter amount of time [4–6]. However, circulating microbubbles have a very short half-life* in vivo* (minutes) and rapidly disappear from the circulation [7]. They are also too large to extravasate from the vascular space to tissue, leading to enhanced heat that occurs only in and around blood vessels [8]. In addition, they can enhance HIFU-mediated heating at multiple points along the beam path, leading to unwanted damage to the tissues proximal to the transducer focus [9].

An alternative to the microbubbles is phase-shift perfluorocarbon (PFC) nanoparticles, which serve as* in situ* cavitation nucleation agents. Under HIFU exposure, the PFC phase is expected to change from liquid to gas form, and a large amount of microbubbles from evaporation can subsequently enhance HIFU thermal effect on the targeted tissues [10–14]. In addition, they have a significantly longer half-life than gas-filled microbubbles in the circulation [15]. As one of PFC compounds, perfluorohexane (PFH) is a temperature-sensitive biocompatible liquid with a boiling point of about 56°C. It can be encapsulated by mesoporous silica nanocapsule (MSNC) to form a nanometer-sized inorganic agent (MSNC-PFH). Wang et al. [12] found that, due to the evaporation from local temperature rise, MSNC-PFH could significantly enhance HIFU thermal ablation. However, it was still unknown whether acoustic cavitation could significantly facilitate the phase transformation of the MSNC-PFH nanoemulsion, leading to the enhancement of HIFU ablation. Using a clinical HIFU device the aim of this study was to investigate whether acoustic cavitation could increase the evaporation of MSNC-PFH and subsequently enhance HIFU ablation in a tissue-mimicking phantom.

## 2. Material and Methods

### 2.1. Phase-Shift Inorganic MSNC-PFH Nanoemulsion

MSNC-PFH nanoemulsion was kindly provided by Professor Hangrong Chen at State Key Laboratory of High Performance Ceramic and Superfine Microstructures, Shanghai Institute of Ceramics, Chinese Academy of Science (Shanghai, China). The preparation and characteristics of MSNC-PFH were previously described in detail [12]. Briefly, it consisted of mesoporous silica nanocapsule as a carrier and perfluorohexane liquid as the inner core. Under electron microscopy the average diameter of the prepared MSNC-PFH nanoemulsion was around 300 nm with mesoporous shell thickness of 50 nm. Dynamic light scattering showed that it had a narrow size distribution with an overall hydrodynamic diameter of 346 nm in water. It was stably and uniformly dispersed in water with the vaporization temperature of around 56°C.

### 2.2. Tissue-Mimicking Phantom

Based on previously described methods [16], an egg white-based, heat-responsive phantom was fabricated in the study. It was nearly transparent at room temperature. When heated up to 60°C, the phantom became a visibly opaque lesion because of the denaturation and coagulation of egg white protein.

The phantom consisted of 15% acrylamide solution (Sigma-Aldrich, St. Louis, MO), 40% egg white, 44.5% degassed deionized water, and 0.5% ammonium persulfate solution (Sigma-Aldrich). After the mixed solution was degassed for 10 min, 0.2 mL MSNC-PFH nanoemulsion was added and then stirred gently to achieve a uniform distribution. The concentration of droplets was 10^7^ droplets/mL in the phantom. In comparison, the same amount of phosphate buffered saline (PBS) was added as a control (PBS-control) without MSNC-PFH nanoemulsion. Finally, 0.15 mL 1,2-bis(dimethylamino)ethane (Sigma-Aldrich) was added to the entire solution to initiate polymerization. The phantom was kept in a 12°C water bath during polymerization period. The dimensions of each phantom used in the experiments were around 6 × 5 × 3.3 cm.

### 2.3. High Intensity Focused Ultrasound System

Experiments were carried out using a clinical CE-approved ultrasound-guided HIFU system (Model JC200, Chongqing Haifu Medical Technology Co., Ltd., Chongqing, China). A diagnostic probe (Esaote, Genoa, Italy) was located in the center of a concave HIFU transducer operating at 0.9 MHz. The integrated transducer can be automatically moved in six directions. The diameter of the HIFU transducer was 220 mm with the focal length of 145 mm. The focal region was ellipsoid, with dimensions of 8 mm along the longitudinal beam axis and 3 mm in the transverse direction.

All exposures were performed at varied duty cycles (DC). The acoustic power delivered for all the experiments was 150 watts, and focal intensity (*I*_SPTA_, spatial-peak temporal-average intensity) was 9750 W/cm^2^. Exposure duration was set to either 5 s or 10 s. The DC of HIFU exposure was 2%, 5%, 10%, and 100%, respectively, at a pulse repetition frequency of 100 Hz. The total energy delivered for each HIFU exposure was shown in Table 1.

### 2.4. Experimental Setup

**Figure 1:**
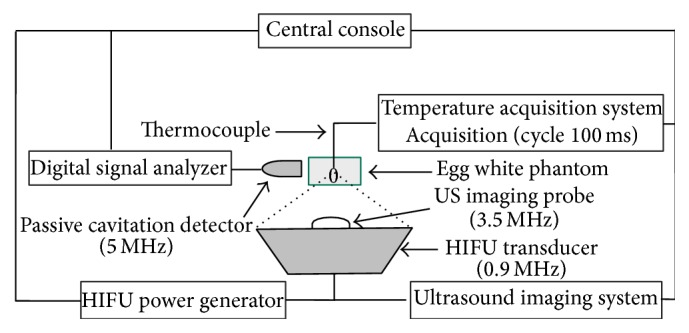
Schematic diagram of the setup for ultrasound-guided high intensity focused ultrasound experiments. The phantom is placed above a 0.9 MHz HIFU transducer with real-time monitoring of ultrasound imaging. Both passive cavitation detector and thermal couple are separately placed around the phantom.

Schematic diagram of the* in vitro* experimental setup was shown in Figure 1. The phantom was immersed in a large tank filled with degassed water and placed above the integrated HIFU transducer and diagnostic probe with real-time monitoring of US imaging. A 0.7 mm needle thermocouple (Omega Engineering Inc., Stamford, Connecticut, USA) was used for temperature measurements during HIFU exposure. It was inserted into the phantom at the focal plane, paralleled to the HIFU beam axis but 0.1 mm off-axis laterally in order to reduce the artifact effect of US vibration on the thermocouple tip. By moving the phantom, the HIFU focus was correctly positioned around the thermocouple tip under US imaging guidance. A low-power HIFU exposure (*P*_ac_ = 30 W, *t* = 1 s) was tested to confirm the exact position of the thermocouple tip until the maximal temperature rise was observed in *x*, *y*, and *z* axes, respectively. During HIFU exposure the temperature change in the phantom was recorded by a temperature data logger (Model FLE5008, Hangzhou Fenle Electronics Co. Ltd., Zhejiang, China).

A passive cavitation detection (PCD) system was used to detect the acoustic emission at the focus of the HIFU transducer. It consisted of a 5 MHz focused transducer (V309, Panametrics, Waltham, MA, USA) and a high-speed digitizer (PXIe-5122, National Instruments, Austin, TX, USA). The aperture of the PCD transducer was 13 mm and focal length was 40 mm with a bandwidth of 3.3–7 MHz at the −6 dB level. In order to detect acoustic emissions from the focus, this transducer was placed perpendicular to and confocal with the HIFU beams. The acoustic emission signals were sampled at 20 MHz rate by the digitizer and finally saved by the computer. LabVIEW software (National Instruments) graphical programming language was used to create the displacement calculation algorithms used in the spectral analysis. Using fast Fourier transform routines, all sampled waveforms were first transformed to the frequency domain. The level of inertial cavitation was then determined by calculating the root mean square (RMS) amplitude of the broadband noise for each spectrum using a method similar to Chen et al. [17]. The calculated RMS amplitude of the broadband noise produced by HIFU exposure was superimposed on the background noise, which could be subtracted as a baseline.

### 2.5. Ultrasound Image Analysis

Real-time US images obtained before and after each HIFU exposure were immediately compared to determine whether a hyperechoic region appeared at the HIFU focus. The hyperechoic region was defined as a region with a distinct increase in the grayscale intensity that was easily observable by a HIFU operator. It was a clinically useful sign indicating the extent of coagulation necrosis. Using HIFU device software, the extent of the bright hyperechoic region at the HIFU focus was determined by the operator, and then the area of the hyperechogenicity was automatically measured. In addition, real-time US imaging videos were recorded during pulsed-HIFU exposure.

### 2.6. Lesion Volume Assessment

Ablation lesions were visible as opaque regions in the transparent phantom. After HIFU ablation, the phantoms were sliced into 1-2 mm along the longitude beam axis, and each ablation lesion was determined by direct visualization and measured with a Vernier caliper. They included the maximal length of the lesion along the longitude beam axis and the maximal width along the perpendicular axis. The volume of lesion was calculated using the formula: Volume = *π* × Maximum  Length × Maximum  Width^2^/6.

### 2.7. Statistical Analysis

SPSS version 17.0 software (SPSS, Chicago, IL, USA) was used for statistical analysis in this study. Data sets were evaluated using one-way analysis of variance (ANOVA), Student's *t*-test, and the least significant difference *t*-test, respectively. All measurement data are expressed as mean values ± standard deviations. At least 6 HIFU exposures were performed for each experimental condition and *P* values less than 0.05 were considered to be statistically significant.

## 3. Results

### 3.1. MSNC-PFH Nanoemulsion Increases the Volume HIFU-Induced Lesion

**Figure 2:**
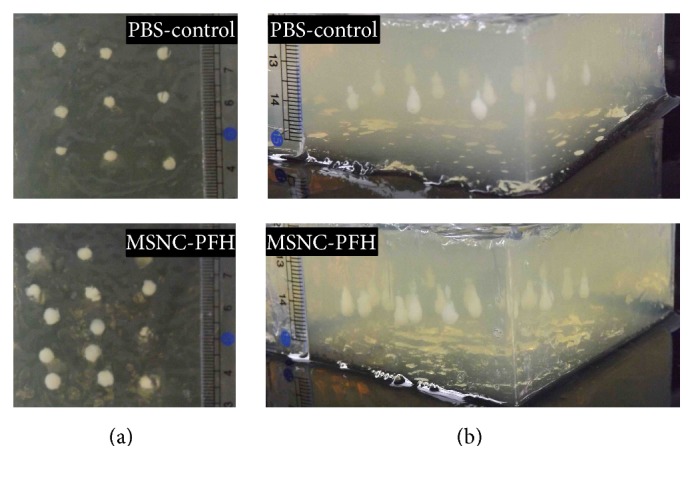
Representative macroscopic images of cross section (b) and longitude-section (a) of lesions induced by 10 s HIFU exposure. In the macroscopic image, an impressive increase in lesion size is observed in the MSNC-PFH phantom while compared to the PBS-control phantom.

**Figure 3:**
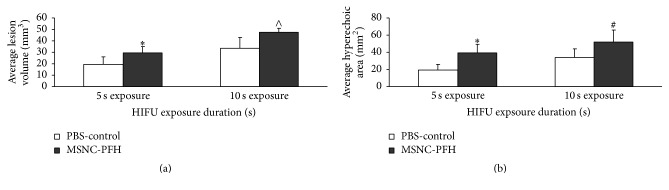
(a) Average volume of HIFU-induced lesions measured by macroscopic examination in the MSNC-PFH and PBS-control phantoms; (b) average area of a bright hyperechoic region at the focus on US imaging immediately after HIFU exposure in the MSNC-PFH and PBS-control phantoms. ^#^ *P* < 0.05 in comparison with the PBS-control group; ^*∗*^ *P* < 0.01 in comparison with the PBS-control group; ^∧^ *P* < 0.001 in comparison with the PBS-control group.

To investigate the effect of MSNC-PFH nanoemulsion on HIFU ablation, HIFU exposures (*P*_ac_ = 150 W, *t* = 5 s or 10 s, DC = 100%) were performed in the MSNC-PFH (*n* = 10) and PBS-control phantoms (*n* = 10), respectively. Representative images of macroscopic lesions induced by 10 s HIFU exposure were shown in Figure 2, including the cross section and longitude-section of the HIFU lesions between the MSNC-PFH and PBS-control phantoms. Compared to the PBS-control, the mean volume of lesions induced by either 5 s or 10 s exposures was significantly larger in the MSNC-PFH phantom (Figure 3(a)). There were statistical differences between the MSNC-PFH and PBS-control phantoms in 5 s exposure (*P* < 0.001) and 10 s exposure (*P* < 0.01). These results demonstrated that MSNC-PFH nanoemulsion could enhance HIFU thermal ablation, resulting in a larger volume of lesions in the phantom.

**Figure 4:**
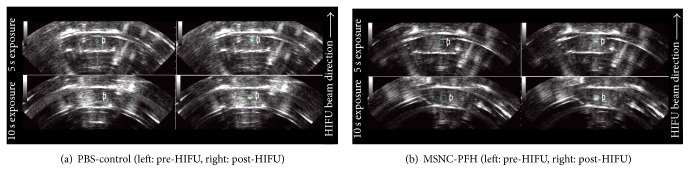
Representative real-time ultrasound images of the MSNC-PFH and PBS-control phantoms before and immediately after 5 s and 10 s HIFU exposure at a duty cycle of 100%. A bright hyperechoic region (arrowhead) is observed immediately after exposure in both MSNC-PFH and PBS-control phantoms.

Real-time B-mode US images were also collected before and immediately after HIFU exposure to determine a hyperechoic area at the HIFU focus between the MSNC-PFH and PBS-control phantoms. Representative images before and immediately after HIFU in both MSNC-PFH and PBS phantoms were shown in Figure 4. A bright hyperechoic region was obviously seen at the HIFU focus on US imaging while compared to the imaging before HIFU. The mean area of the hyperechogenicity in the MSNC-PFH phantom was significantly larger than that in the PBS-control phantom. There were statistical differences between them in 5 s exposure (*P* < 0.01) and in 10 s exposure (*P* < 0.05), as shown in Figure 3(b).

### 3.2. Real-Time US Imaging during HIFU Exposure

**Figure 5:**
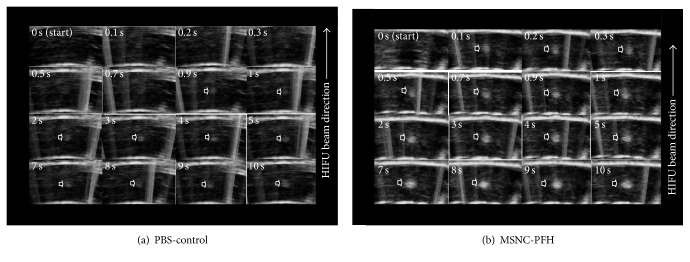
Representative real-time ultrasound images of time evolution of a hyperechoic region (arrowhead) with 10 s HIFU exposure at a duty cycle of 2% in the MSNC-PFH and PBS-control phantoms. (a) Hyperechoic changes at the HIFU focus in the PBS-control phantom: a bright hyperechoic region occurs on the US imaging at 0.9 s after HIFU exposure, with expanded views of the region of the HIFU lesion (from 1 s to 10 s). (b) Hyperechoic changes at the HIFU focus in the MSNC-PFH phantom: a bright hyperechoic region occurs on the US imaging at 0.1 s after HIFU exposure, with expanded views of the region of the HIFU lesion (from 0.2 s to 10 s). During pulsed-HIFU exposure, the hyperechoic region is observed growing and migrating towards the HIFU transducer in both MSNC-PFH and PBS-control phantoms.

In order to reduce the interference of HIFU with the imaging system, pulsed-HIFU exposure was used to help the recording of real-time US imaging videos. HIFU exposures with 2% DC (*P*_ac_ = 150 W, *t* = 10 s) were performed to determine when the hyperechogenicity occurred at the HIFU focus in both MSNC-PFH (*n* = 6) and PBS phantoms (*n* = 6). As shown in Figure 5, a bright hyperechoic region occurred on the US imaging at 0.1 s after HIFU exposure in the MSNC-PFH phantom, whereas the hyperechogenicity occurred at 0.9 s after HIFU exposure in the PBS-control phantom. Subsequently, the hyperechoic region was observed growing and migrating towards the HIFU transducer. The results indicated that the first few pulsed-HIFU could deliver energy enough to vaporize MSNC-PFH droplets at the focus, suggesting that acoustic cavitation rather than heat might initiate the vaporization of the nanoemulsion.

### 3.3. Temperature Changes during HIFU Exposure

**Figure 6:**
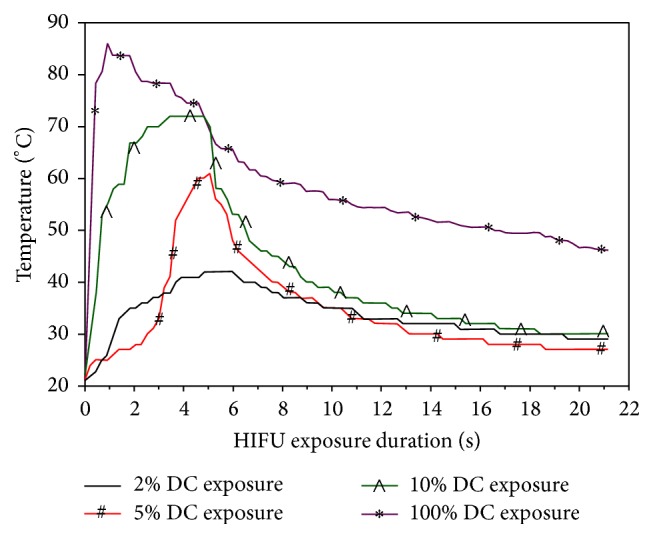
Temperature elevations measured at the HIFU focus as a function of time during a 5 s HIFU exposure at the DC of 2%, 5%, 10%, and 100% in the MSNC-PFH phantom. The peak temperature that is higher than the evaporation temperature of PFH (56°C) is observed in HIFU exposures at the duty cycle of 5%, 10%, and 100%, respectively.

To quantify the amount of HIFU-mediated heating in the MSNC-PFH phantom, temperature measurements were performed during HIFU exposure. The DC selected for HIFU exposure (*P*_ac_ = 150 W, *t* = 5 s) was 2% (*n* = 6), 5% (*n* = 6), 10% (*n* = 6), and 100% (*n* = 6), respectively, and the temperature was calculated at multiple time points. The relative differences in temperature rise in the MSNC-PFH phantom were shown in Figure 6 as a function of time during and after HIFU exposures at varied duty cycles. The average peak temperatures measured at the HIFU focus were about 78.16 ± 5.64°C at a DC of 100%, 70.17 ± 6.43°C at 10%, 53.17 ± 4.54°C at 5%, and 42.00 ± 5.55°C at 2%, respectively. In addition, the time required for the peak temperature rise was significantly different. When DC was 100%, the time required for it was only 1 s. However, in the remaining 3 pulsed-HIFU exposures the required time was about 5 s, which was 5 times longer than the HIFU exposure at a DC of 100%. The results revealed that HIFU exposures with higher DC could have a stronger effect on the heat accumulation in the MSNC-PFH phantom.

### 3.4. Acoustic Cavitation Enhances HIFU Ablation in MSNC-PFH Phantom

**Figure 7:**
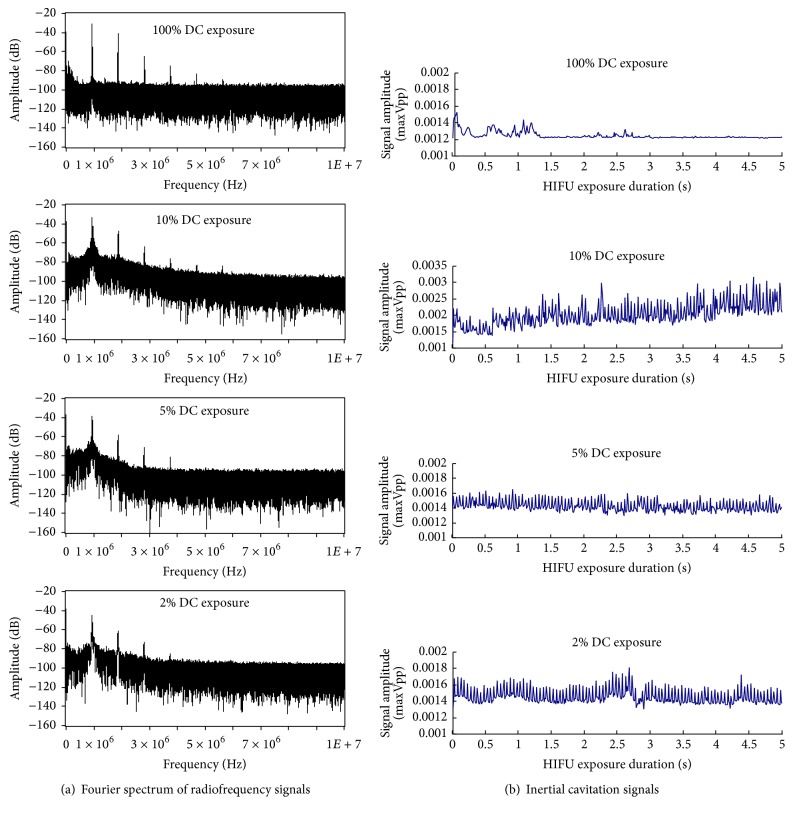
Representative images of Fourier spectra of the radiofrequency signals (a) and typical time evolutions of inertial cavitation activity as a function of time for PCD signals (b) at the HIFU focus during exposures at the DC of 2%, 5%, 10%, and 100% in the MSNC-PFH phantom.

To investigate the effect of inertial cavitation on the acoustic vaporization of the MSNC-PFH nanoemulsion, a PCD method was used to monitor the activities of inertial cavitation at the HIFU focus during HIFU exposure (*P*_ac_ = 150 W, *t* = 5 s) at the varied duty cycles. The DC selected for HIFU exposure (*P*_ac_ = 150 W, *t* = 5 s) was 2% (*n* = 10), 5% (*n* = 10), 10% (*n* = 10), and 100% (*n* = 10), respectively.  Figure 7 showed typical Fourier spectra of the radiofrequency signals and typical time evolutions of inertial cavitation activity as a function of time for PCD signals at the HIFU focus during exposures at the DC of 2%, 5%, 10%, and 100%. In the Fourier spectrum of radiofrequency signals broadband noise was interpreted as inertial cavitation, and subharmonic noise was read as stable cavitation. As shown in Figure 7(a), significant increases were observed in the level of both broadband and subharmonic noises during HIFU exposures. Erratic changes with respect to rise and fall in amplitude of inertial cavitation level were also seen, with an overall increase in level of cavitation during HIFU exposures at the various DC (Figure 7(b)). However, our results showed that inertial cavitation was much stronger in the pulsed-HIFU than that in the continuous-wave (100% DC) HIFU exposure. Among them, the strongest cavitation activity was observed at a DC of 10%.

**Figure 8:**
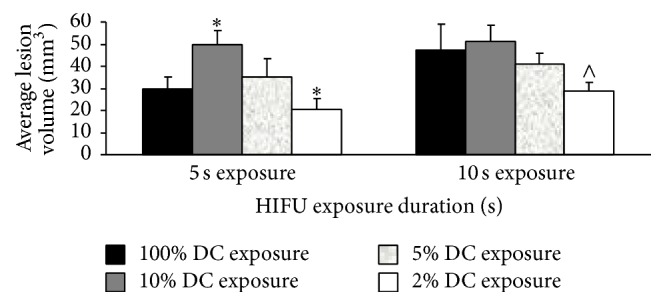
Average volume of lesions measured by macroscopic examination after HIFU exposures at the DC of 2%, 5%, 10%, and 100% in the MSNC-PFH phantom. ^*∗*^ *P* < 0.005 in comparison with 100% DC HIFU exposure; ^∧^ *P* < 0.001 in comparison with 100% DC HIFU exposure.

Subsequently, the mean volume of lesions was measured by macroscopic examination in the MSNC-PFH phantom after HIFU exposures (*P*_ac_ = 150 W, *t* = 5 s or 10 s) at the various DC. As shown in Figure 8, the mean volumes of lesion were directly related to the DC of HIFU exposure. For both 5 s and 10 s HIFU exposures, the mean lesion volumes induced by HIFU exposure at the DC of 100%, 10%, 5%, and 2% were 29.55 ± 5.51 and 47.48 ± 11.69, 49.76 ± 6.12 and 50.98 ± 7.61, 35.36 ± 8.28 and 41.22 ± 4.67, and 20.38 ± 4.77 and 28.65 ± 4.10, respectively. Compared to the exposure at a DC of 100%, the mean volume of lesion at a DC of 2% was significantly smaller in 5 s exposure (*P* < 0.005) and 10 s exposure (*P* < 0.001). However, the mean volume of lesion induced by 5 s exposure at a DC of 10% was significantly larger than that at a DC of 100% (*P* < 0.005). No significant difference of the lesion volume in 5 s and 10 s exposure was observed between the exposures at the DC of 100% and 5% (*P* > 0.05). The results revealed that acoustic cavitation delivered by HIFU exposure at a DC of 10% could significantly increase the vaporization of MSNC-PFH droplets, resulting in stronger cavitation-enhanced HIFU ablation. However, our study also showed that this effect was significantly limited in HIFU exposure at a DC of 2%, suggesting that there might be the threshold of cavitation activity for the vaporization of MSNC-PFH nanoemulsion.

## 4. Discussion and Conclusion

Acoustic droplet vaporization (ADV) is a recently exploited phenomenon in which a liquid droplet is induced by cyclic pressure waveforms (acoustic waves) to form a vapor phase [18]. Previous studies showed that phase-shift perfluorocarbon nanoparticles could be vaporized by focused US to nucleate inertial cavitation and subsequently enhance HIFU-mediated heating [10–14]. In this study a novel phase-shift inorganic nanoemulsion is used to enhance HIFU ablation. It has a temperature-sensitive PFH core that is encapsulated within the hollow interior of MSNC through mesopores to produce a MSNC-PFH aqueous solution with extraordinarily high thermal and chemical stability [19]. Our results show that, compared to the PBS-control, the use of MSNC–PFH nanoemulsion can significantly increase the volume of lesion by enhancing local energy deposition at the acoustic focus. There are no heating effects and protein denaturation in the off-focus regions including the pre- and postfocal US propagation pathway. Our results have demonstrated that as a nanoemulsion droplet MSNC-PFH can enhance HIFU thermal ablation* in vitro*.

Real-time US imaging is used in the study to guide HIFU ablation. Our results confirm the findings obtained from previous studies that US imaging could visualize the ADV microbubbles as a hyperechoic region at the acoustic focus after HIFU exposure [10, 20]. In order to reduce the interference of HIFU device with the imaging system, pulsed-HIFU irradiation at a DC of 2% was also performed in the study to observe the process of ADV and the subsequent formation of the lesion during the pulse off-time on US imaging. Our recorded videos showed that microbubbles occurred within the focus at 0.1 s after HIFU in the MSNC-PFH phantom whereas ADV occurred at 0.9 s in the PBS-control, indicating that acoustic cavitation rather than heat could initiate the ADV process that occurred much earlier than the formation of HIFU lesion. In addition, we found that US imaging could in real-time visualize the hyperechoic region, its growing and migrating process along the longitudinal beam axis towards the transducer during pulsed-HIFU exposure. As there is a good correspondence between the size of the hyperechoic region and HIFU lesion [10], this is an important finding that could establish an imaging feedback method for the control of ADV process and the prediction of the lesion formation at the acoustic focus during HIFU exposure. In order to sufficiently ablate a targeted tumor, HIFU treatment needs constant monitoring and adjustment of acoustic power and exposure duration. Our results have revealed that, in addition to guiding cavitation-enhanced HIFU procedure, real-time B-mode US imaging can become an important feedback-controlled tool for monitoring the lesion formation and adjusting the acoustic power during pulsed-HIFU exposure.

There are two major destructive effects on targeted tissues during HIFU ablation. One involves high time-average intensity to make use of heat for thermal ablation [21]. The other concerns high pulse-average intensity with low duty cycle for the cavitation effect while reducing heating [22, 23]. In this study the peak temperature and PCD signal were measured for HIFU exposures at different DC to determine which effect dominates the ADV process and lesion formation in the MSNC-PFH phantom. We found that at the same acoustic power (*P*_ac_ = 150 W) and exposure duration (*t* = 5 s), DC could be an important factor to affect the temperature rise and inertial cavitation at the HIFU focus. When DC was 100%, the highest peak temperature (78.16 ± 5.64°C) was observed whereas the inertial cavitation activity was the weakest, suggesting the temperature rise up to above the evaporation temperature of PHF could play an important role in HIFU thermal ablation. In the pulsed-HIFU exposures, the average peak temperature was 70.17 ± 6.43°C at a DC of 10% and 53.17 ± 4.54°C at 5%, but the lowest peak temperature (42.00 ± 5.55°C) was observed at a DC of 2%. In addition, inertial cavitation was much stronger in the pulsed-HIFU than that in the continuous-wave HIFU, with the strongest one occurring at a DC of 10%. These results reveal that DC can significantly influence heat accumulation and inertial cavitation in the MSNC-PFH phantom. The higher the duty cycle is, the easier the heat deposition can locally accumulate. Continuous-wave HIFU exposure can have a significant effect on the heat accumulation with a low cavitation activity. In contrast, if DC is too low, inertial cavitation becomes the most important factor to trigger the ADV process and lesion formation in the MSNC–PFH phantom as the local accumulation of heat is limited.

On the other hand, we found that DC could directly influence the volume of HIFU-induced lesion and total US energy required for ablation in the MSNC-PFH phantom. For both 5 s and 10 s HIFU exposures, the largest lesion volume was observed in 10% DC HIFU exposure. There was significant difference of the lesion volume between 5 s continuous-wave (100% DC) and 10% DC HIFU exposures. As the peak temperature at a DC of 10% is higher than the vaporization temperature of PFH, these results have indicated that both inertial cavitation and heat could significantly increase the ADV of MSNC-PFH droplets, leading to stronger cavitation-enhanced HIFU ablation. In addition, compared to continuous-wave HIFU, 5 s HIFU exposure at a DC of 10% can reduce total US energy required for ablation from 750 J to 75 J, as shown in Table 1. These demonstrate that pulsed-HIFU exposure at a DC of 10% can significantly reduce total US energy required for MSNC-PFH enhanced ablation and treatment time, as well as increasing the volume of lesion. However, when the DC decreases to 2%, the thermal effect is limited and only cavitation-enhanced HIFU ablation occurs, resulting in the smaller lesion volume in the MSNC-PFH phantom.

The long-term goal of using nanodroplets is to reduce US energy and treatment time required to ablate solid tumors, as well as improve the safety of HIFU in clinical applications. Our results demonstrate that using a clinical HIFU device it is possible to vaporize MSNC-PFH nanoemulsions* in vitro* at low duty cycle. However, there are some limitations in the study. The gel phantom is not as attenuate as solid tumors and with no blood perfusion; these will certainly influence the amount of US energy required for HIFU ablation. In addition, the peak temperature measured by a single thermocouple cannot represent the spatial distribution of heating at the focus.

In conclusion, acoustic cavitation can significantly increase the vaporization of MSNC-PFH nanoemulsions and subsequently enhance HIFU thermal ablation in the thermosensitive phantom. Appropriate pulsed-HIFU exposure can not only significantly increase the volume of lesion but also reduce total US energy required for MSNC-PFH nanoemulsion-mediated HIFU thermal ablation. However, further studies are needed to investigate the enhanced effects of MSNC-PFH nanoemulsion on HIFU thermal ablation in animal tumor models.

## Figures and Tables

**Table 1:** Total ultrasound energy delivered for HIFU exposures at varied duty cycles.

Duty cycles	Acoustic power (watts)	Exposure duration (second)		Total ultrasound energy (joules)	
2% exposure	150	5	10	15 (5 s)	30 (10 s)
5% exposure	150	5	10	37.5 (5 s)	75 (10 s)
10% exposure	150	5	10	75 (5 s)	150 (10 s)
100% exposure	150	5	10	750 (5 s)	1500 (10 s)
